# Neurological deterioration in a patient with HIV-associated cryptococcal meningitis initially improving on antifungal treatment: a case report of coincidental racemose neurocysticercosis

**DOI:** 10.1186/s12879-021-06425-9

**Published:** 2021-07-31

**Authors:** Newton Kalata, Jayne Ellis, Laura Benjamin, Samuel Kampondeni, Peter Chiodini, Thomas Harrison, David G. Lalloo, Robert S. Heyderman

**Affiliations:** 1 Malawi Liverpool Wellcome Trust Clinical Research Programme, Liverpool, UK; 2 Internal Medicine Department, College of Medicine University of Malawi, Malawi, UK; 3 Hospital for Tropical Diseases, UCLH, London, UK; 4 Stroke Research Centre, Institute of Neurology, University College London, London, UK; 5 Brain Infection Group, Institute of Infection and Global Health, University of Liverpool, Liverpool, UK; 6 Division of Infectious Disease Immunology, St George Hospital, London, UK; 7 Clinical Sciences and International Public Health, Liverpool School of Tropical Medicine, Liverpool, UK; 8 Division of Infection and Immunity, University College London, London, UK

**Keywords:** HIV, Cryptococcal meningitis, Racemose neurocysticercosis

## Abstract

**Background:**

Managing HIV-associated cryptococcal meningitis (CM) can become challenging in the presence of concurrent unusual central nervous system infections.

**Case presentation:**

A 58-year old HIV infected woman new ART starter, who was being treated effectively for cryptococcal meningitis, represented with worsening of neurological symptoms. Brain MRI revealed a multicystic lesion in the left temporal lobe. Anti-fungal treatment was escalated for a suspected cryptococcoma, but post-mortem CSF serological test confirmed racemose neurocysticercosis.

**Conclusion:**

Patients with HIV-associated CM are highly immunocompromised and may have multiple pathologies simultaneously. In endemic countries, neurocysticercosis should be considered in the differential diagnosis where there is central nervous system deterioration despite effective therapy for CM.

## Background

Cryptococcal meningitis (CM) is the most common neurological complication of advanced immunodeficiency in HIV-infected individuals. In 2014, 250,000 incident cases were reported and CM accounted for 15% of AIDS-related deaths [1]. With current antifungal treatments, mortality attributable to CM ranges between 20 and 50% [1, 2]. Despite the scale-up of ART coverage in sub-Saharan Africa, the numbers of people progressing to advanced immunosuppression exceed the capacity of ART programs [3]. Patients with HIV-associated CM remain at risk of other opportunistic infections. Managing CM in HIV-infected patients can become challenging when other unusual central nervous system infections occur concurrently. We describe a case of coincidental racemose neurocysticercosis in a patient being treated for HIV-associated cryptococcal meningitis, clinically and microbiologically responding to antifungal therapy.

## Case presentation

A 58-year old HIV-infected woman presented with a 3-day history of headache to Queen Elizabeth.

Central Hospital (QECH), a teaching hospital in Blantyre, Malawi. The headache was associated with fever, vomiting, confusion, and loss of consciousness a day before presentation. She did not report any convulsions or constitutional symptoms. She had started first-line ART (Tenofovir, Lamivudine and Efavirenz) two months before presentation and reported good adherence to treatment. The patient was afebrile and her Glasgow coma scale (GCS) was 14/15. She had marked neck stiffness, there was no evidence of focal neurological deficit. Her baseline CD4 count was 26 cells/μl and serum glucose was 6.1 mmol/L (normal 4.0 to 7.0 mmol/L). CSF showed an opening pressure of 23cmH2O (normal range 5 to 15 cmH2O) [4], was positive on a lateral flow assay (LFA) (IMMY) for cryptococcal antigen (crAg), white cell count (WBC) 0 cells/mm3 (normal < 5), red cell count (RCC) 160 cells/mm3 (normal < 10), protein 3.01 g/L (normal 20–40), glucose 2.2 mmol/L (normal range 2.5–4.5). Quantitative cryptococcal culture (QCC) subsequently demonstrated 910 colony-forming units (CFU) per ml. A combination of oral fluconazole 1200 mg once a day and flucytosine 1000 mg 6 hourly was commenced as part of the ACTA clinical trial (Open-label, multicentre randomized trial; doi: 10.1056/NEJMoa1710922). She showed a good clinical response to antifungal treatment with resolution of confusion and vomiting but required daily lumbar punctures (LP) for persistent raised intracranial pressure (ICP). By day 14 her clinical symptoms had improved, her ICP normalized and there was no growth on CSF fungal culture. She was commenced on consolidation therapy with fluconazole 800 mg once daily alone and discharged with a scheduled review in two weeks.

**Fig. 1:**
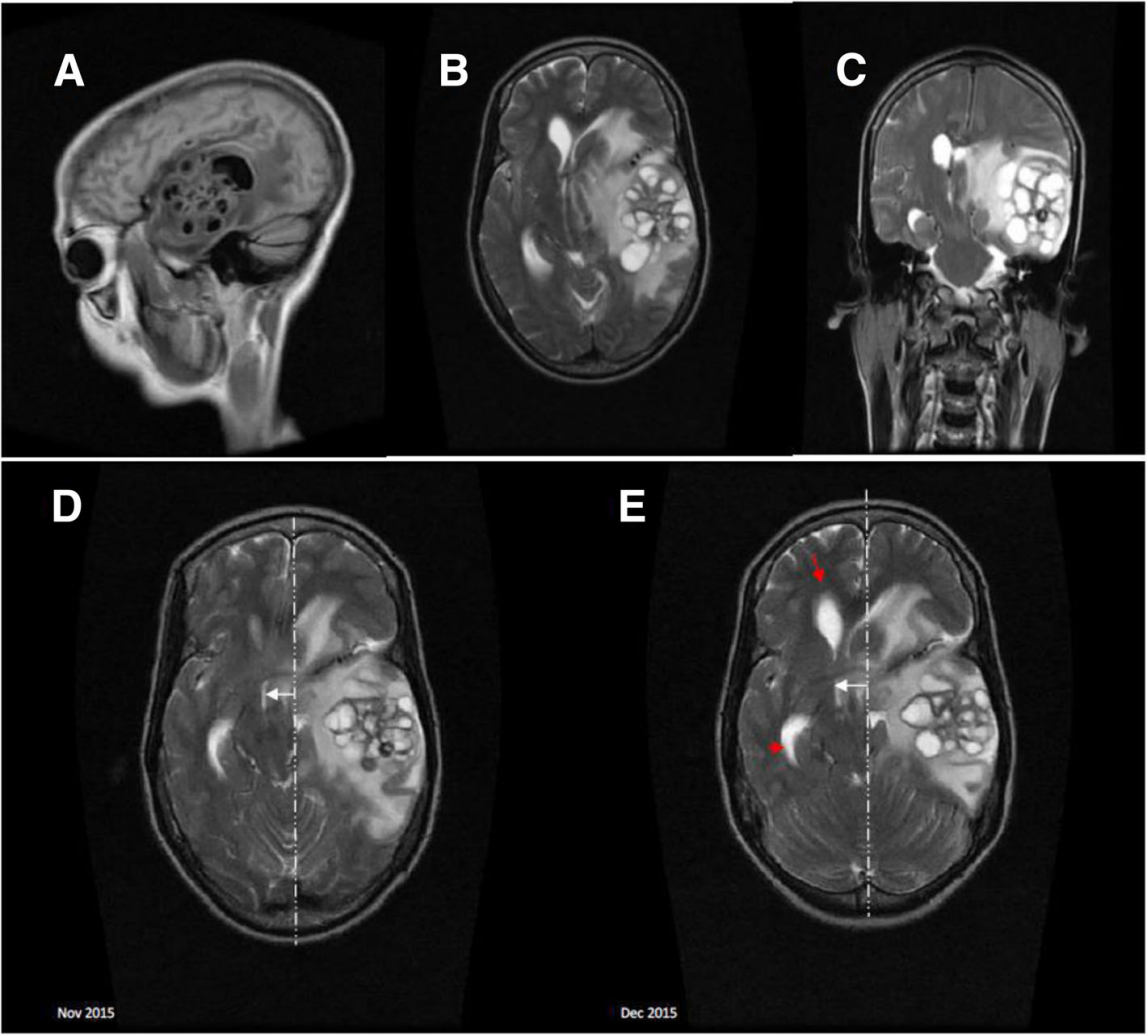
Sagittal T1 Fluid-attenuated inversion recovery (**A**), Axial T2 (**B**), and Coronal T2 images (**C**) were taken 4 weeks after the initial presentation: showing a 6cmx5cmx6cm multicystic mass lesion in the left temporal lobe with surrounding oedema. It is compressing the lateral ventricles, causing early hydrocephalus and midline shift to the right. Axial T2 were taken 4 weeks after the initial presentation (**D**), and 14 days after retreatment with antifungals and steroids (**E**): The multicystic mass in the left temporal lobe had increased in size (**B**) with more surrounding oedema, progressing obstructive hydrocephalus (**B**; red arrows), increased midline shift, effacement of basilar cisterns and compression of the brainstem

28 days after her initial presentation, she represented with headache, confusion, and neck pain. She had a new right-sided upper motor facial nerve palsy. Lumbar puncture (LP) showed a raised ICP of 28 cmH2O. Serum glucose was 5.2 mmol/L and CSF analysis showed WBC 0 cells/mm3, RCC 130 cells/mm3, glucose 2.2 mmol/L, protein 2.61 g/L, and was still positive for crAg (titers were not available). The QCC, available after five days, was 700 cfu/ml (previously sterile) and a repeat CD4 count was 49 cells/μl. Magnetic resonance imaging (MRI) of her brain demonstrated a 6 cm × 5 cm × 6 cm multicystic mass lesion in the left temporal lobe with surrounding edema that was compressing the lateral ventricles causing early hydrocephalus and a 1 cm midline shift to the right (Fig. 1 A- D). Thigh radiographs did not show any evidence of spindle-shaped calcification. She was treated for suspected CM relapse and cryptococcoma with intravenous amphotericin B deoxycholate 1 mg/kg and oral fluconazole 1200 mg daily for 14 days according to the Malawi Standard Treatment guidelines, plus prednisolone 60 mg once daily to reduce the cerebral edema. She was monitored closely as an inpatient and samples were sent for neurocysticercosis serological testing in London as they were not available in Malawi.

After 14 days of treatment, a follow-up MRI of the brain demonstrated worsening radiological changes: the mass had increased by 200%, increased edema, increased midline shift to the right, more obstructive hydrocephalus of the right ventricle, more pronounced effacement of basilar cisterns, and compression on the brainstem (Fig. 1D). Clinically, however, the patient’s confusion and headache improved and she was discharged home on prednisolone and fluconazole consolidation therapy. The patient died unexpectedly a week later at home. Post-mortem, a neurocysticercosis enzyme-linked immunoelectrotransfer blot (EITB) assay was positive for cysticercosis on CSF but negative on serum. It was performed in the Department of Clinical Parasitology, Hospital for Tropical Diseases London.

## Discussion and conclusion

This case demonstrates the difficulties of managing CM in limited resource settings and the diagnostic challenges encountered when a patient with known CM presents with neurological deterioration late in the course of treatment. These severely immunocompromised patients are at risk of treatment failure and paradoxical immune reconstitution inflammatory (IRIS) reactions. However, they are also vulnerable to a range of other opportunistic CNS infections that form important differential diagnoses when neurological deterioration is observed despite effective treatment.

CM paradoxical IRIS is a key differential diagnosis. It is suspected when a patient who initially responded to antifungal therapy clinically deteriorates. The cause is an exaggerated inflammatory response that occurs due to the recovery of disease-specific immune response after ART initiation. The incidence of CM paradoxical IRIS is between 13 and 30% and tends to occur 1–2 months post ART initiatio n[5]. .Our patient presented with neurological deterioration 28 days after commencement of anti-fungal therapy and approximately 3 months after starting ART. A diagnosis of paradoxical IRIS was considered, but was thought to be less likely because a repeat CD4 count did not suggest rapid immune reconstitution, and CSF analysis showed no signs of inflammation [5].

Instead, the positive cryptococcal CSF culture prompted us to treat for suspected cryptococcoma formation with anti-fungal treatment escalation and prednisolone. Cryptococcoma are granulomatous lesions that form around a focal tissue infection of *Cryptococcus neoformans*.

Typically, the formation of cryptococcoma depends on an inflammatory response and are therefore more often seen in immunocompetent individuals or those who have initiated on ART. Case reports of cryptococcoma have described patients who presented with persistent neurological findings with sterile CSF or a low fungal burden [6]. This is because the walled off mass is thought to continuously shed *Cryptococcus neoformans* into the CSF [6]. As with CM, the mainstay of treatment is combination amphotericin-based antifungal agents.

Cysticercosis is a human parasitic infection caused by larval cysts of the tapeworm *Taenia solium*. It is commonly found in rural areas of low and middle-income countries with poor sanitation. Human ingestion of *T. solium* eggs releases larvae which migrate from the intestine, spread through the body via the bloodstream and develop into cysts in a variety of sites. The most common sites include the muscles, eyes, brain, and spinal cord. Presenting symptoms depend on the location, size, number, and stage of the cyst [7]. According to community based human prevalence studies, sero-prevalence of human cysticercosis ranges from 7 to 22% in Africa [8]. Neurocysticercosis (NCC) manifests with nonspecific symptoms and lack of diagnostic tests makes definitive diagnosis particularly difficult in this setting. A systematic review of clinical manifestations of NCC found that nearly 80% of patients with NCC had late onset seizures, 38% had headaches and 12% had raised intracranial pressure [9]. Our patient had no history of seizures; the subsequent headache, raised ICP and cognitive decline was initially thought to be related to her CM. NCC was considered as a possible diagnosis but given its relatively low incidence rate of about 5.5% in the region [8], a decision was made to treat our patient for CM associated cryptococcoma while investigating her further.

Serological confirmation of NCC in HIV-infected individuals is problematic. We have found no robust analyses of serology in the context of HIV and NCC in the literature. The positive CSF serology without detectable serum antibody is unexplained. It could be due to local antibody production in the context of insufficient antigen exposure peripherally, however we have no evidence for this.

Brain imaging is not routinely available at presentation for patients with meningitis due to financial constraints in Malawi. It is, therefore, reserved for patients who deteriorate despite appropriate treatment or who develop complications just like our patient. The imaging appearance of the cystic lesions was consistent with a rare extra-parenchymal form of NCC called racemose NCC. This form derives its name from the grape-like appearance of the confluent cysts on MRI which are located in the basal subarachnoid spaces or the Sylvian fissure [10]. The confluent cysts result from excessive and unusual growth of the cyst wall and development of vesicular lesions without scoleces. The primary effect of growth of the racemose cysts is to cause intracranial hypertension and hydrocephalus by obstructing the flow of CSF. This makes prognosis worse except when neurosurgery is available [10]. In our patient, the neuroimaging findings of multiple cystic lesions without a discernable scolex and asymmetrical early hydrocephalus, and the detected cysticercal antibody fulfill two major neuroimaging and one major clinical criteria consistent with a definitive diagnosis of NCC according to the revised diagnostic criteria for NCC [11].

Treatment of NCC involves anthelminthic treatment, corticosteroids and management of complications like seizures, headaches, and hydrocephalus. For parenchymal NCC, randomized clinical trials have shown that use of combined antiparasitic therapy with albendazole and praziquantel increases the parasiticidal effect in patients with multiple brain cysts without increased side effects, but the trial did not include racemose NCC cases [12].

Patients with HIV-associated CM are profoundly immunocompromised and may have multiple pathologies simultaneously. Given the paucity of access to neuroimaging facilities and serological assays in many African countries, this condition may be more frequent than generally thought. In endemic areas, clinicians should be aware of neurocysticercosis and its unusual manifestations in HIV-infected patients. Where there is CNS deterioration despite effective therapy for CM or other CNS opportunistic infections, neurocysticercosis should be included in the differential diagnosis. Empirical treatment without diagnostic brain imaging is not recommended but symptom control with appropriate anticonvulsant therapy is a priority. Corticosteroids in addition to specific antiparasitic therapy may be necessary where there is lesion associated edema on imaging.

## Data Availability

The original data used in the presentation of this case report are available from the corresponding author on reasonable request.

## Abbreviations

ART: Anti-retroviral therapy
CFU: Colony forming units
CM: Cryptococcal meningitis
CSF: Cerebrospinal fluid
EITB: Enzyme-linked immunoelectrotransfer blot
GCS: Glasgow coma score
HIV: Human Immunodeficiency Virus
ICP: Intracranial pressure
IRIS: Immune reconstitution inflammatory syndrome
LFA: Lateral flow assay
LP: Lumber puncture
MRI: Magnetic resonance imaging
NCC: Neurocysticercosis
RCC: Red cell count
QCC: Quantitative cryptococcal culture
QECH: Queen Elizabeth Central Hospital
WCC: White cell count
